# Dynamics and asymmetry in the dimer of the norovirus major capsid protein

**DOI:** 10.1371/journal.pone.0182056

**Published:** 2017-07-27

**Authors:** Thibault Tubiana, Yves Boulard, Stéphane Bressanelli

**Affiliations:** Institute for Integrative Biology of the Cell (I2BC), CEA, CNRS, Univ Paris Sud, Université Paris-Saclay, Gif sur Yvette cedex, France; Friedrich-Alexander-Universitat Erlangen-Nurnberg, GERMANY

## Abstract

Noroviruses are the major cause of non-bacterial acute gastroenteritis in humans and livestock worldwide, despite being physically among the simplest animal viruses. The icosahedral capsid encasing the norovirus RNA genome is made of 90 dimers of a single *ca* 60-kDa polypeptide chain, VP1, arranged with T = 3 icosahedral symmetry. Here we study the conformational dynamics of this main building block of the norovirus capsid. We use molecular modeling and all-atom molecular dynamics simulations of the VP1 dimer for two genogroups with 50% sequence identity. We focus on the two points of flexibility in VP1 known from the crystal structure of the genogroup I (GI, human) capsid and from subsequent cryo-electron microscopy work on the GII capsid (also human). First, with a homology model of the GIII (bovine) VP1 dimer subjected to simulated annealing then classical molecular dynamics simulations, we show that the N-terminal arm conformation seen in the GI crystal structure is also favored in GIII VP1 but depends on the protonation state of critical residues. Second, simulations of the GI dimer show that the VP1 spike domain will not keep the position found in the GII electron microscopy work. Our main finding is a consistent propensity of the VP1 dimer to assume prominently asymmetric conformations. In order to probe this result, we obtain new SAXS data on GI VP1 dimers. These data are not interpretable as a population of symmetric dimers, but readily modeled by a highly asymmetric dimer. We go on to discuss possible implications of spontaneously asymmetric conformations in the successive steps of norovirus capsid assembly. Our work brings new lights on the surprising conformational range encoded in the norovirus major capsid protein.

## Introduction

**Fig 1:**
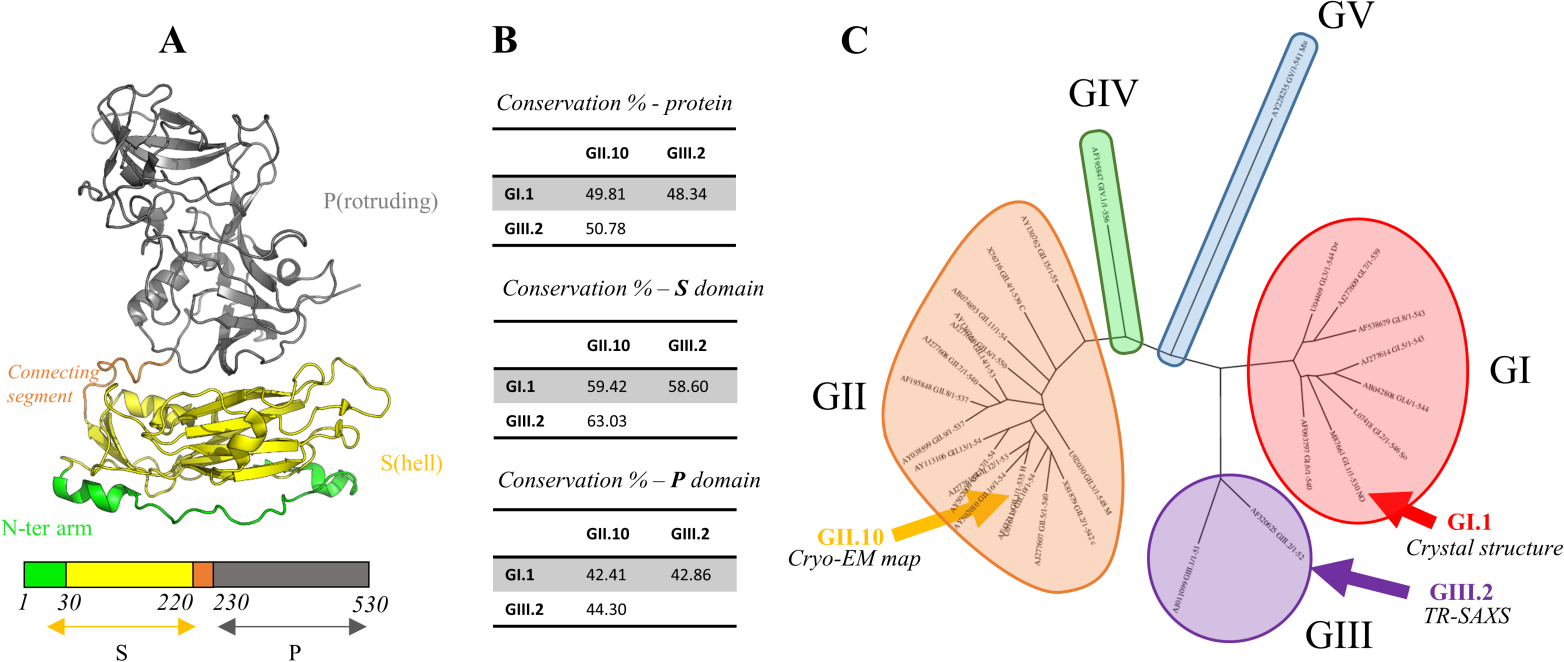
Crystal structure and sequence diversity of the norovirus VP1 capsid protein. (A) Structure of the norovirus major capsid protein (VP1). The N-terminal S domain is in yellow and the C-terminal P domain in grey. They are separated by a connecting segment of approximately 10 amino acids (orange). The N-terminal arm of the S domain is colored in green. Boundaries of domains are indicated between the primary structure diagram. (B) Pairwise sequence identities between VP1 for GI.1 (crystal structure), GII.10 (Cryo-EM Map) and GIII.2 (TR-SAXS) and between their P and S domains. (C) Dendrogram of 29 representative VP1 sequences taken from Zheng et al. [6]. The 29 genotypes' accession numbers are given in the "Materials and Methods" section. The genotypes are grouped in the five major norovirus genogroups GI to GV (colored circles). Capsids for which structural data are available (GI.1, GII.10 and GIII.2) are indicated by arrows.

Noroviruses are major causes of acute gastroenteritis worldwide [1]. They are extremely resilient and biodisponible, explaining in part their high infectivity by the fecal-oral route. The norovirus virion's major protein component comprises 180 copies of a single polypeptide chain (viral protein 1, or VP1) that together encase the *ca* 7500-base single-stranded RNA genome and a few copies of viral protein 2 (VP2). Upon production in the baculovirus system, VP1 self-assembles into empty virus-like particles (VLP) that are antigenically and structurally indistinguishable from infectious particles collected in infected individuals' stools [2]. This has allowed early biochemical studies despite the difficulty in growing noroviruses in cell culture and is still the method of choice to study VP1. Indeed, the only atomic model of VP1 has been obtained for such a VLP with the crystal structure of the Norwalk virus capsid [3]. This work showed that in the particle the 180 copies of VP1 are arranged with classical T = 3 icosahedral symmetry [4]. There are thus 60 identical asymmetric units, each containing three VP1 molecules A, B and C with quasi-equivalent contacts to their neighbors. VP1 has a two-domain structure (Fig 1A), with an N-terminal shell domain (S) making up the spherical protein capsule and a C-terminal protruding domain (P) making up 90 dimeric spikes on the exterior surface of the shell. Indeed the large contact surface between pairs of P indicates that the capsid building block is a VP1 dimer. The segment connecting S to P and an arm N-terminal to S allow the small conformational adjustments that differentiate quasi-equivalent dimers A-B and C-C. Contacts between the two P domains provide most of the dimeric interaction surface but S domains also interact across the two-fold axis in both types of dimers, as remarked by Prasad et al. [3].

As with most RNA viruses there is a high diversity of noroviruses. They are classified in at least five genogroups, each genogroup harboring several genotypes [5]. Typically pairwise sequence identity between VP1 from distinct genogroups is about 50% (Fig 1B).

Norwalk virus, for which the VLP crystal structure is known, belongs to genotype 1 of genogroup I (GI.1, Fig 1C). Structural data for other genogroups includes cryo-electron microscopy (cryo-EM) for GV infectious virion [7] and GII VLP [8]. Those works showed a distinct conformation compared to the GI crystal structure of the 90 P dimers, that are raised off the shell and rotated, forming a cagelike structure around the S shell. Finally, our own work by time-resolved small-angle X-ray scattering (TR-SAXS) on a GIII VLP showed that after *in vitro* dissociation of the VLP into its constituting dimers, reassembly proceeds through long-lived, elongated intermediates made of 10–11 dimers [9]. Interestingly, we found then that the dissociated GIII VP1 dimer has a much larger radius of gyration than would be expected from the crystal structure of the GI dimers in the VLP. In the present work, we further explore the conformations and dynamics of the norovirus VP1 dimer by molecular modeling and molecular dynamics simulations and with additional SAXS data on the GI dimer.

## Materials and methods

### Homology modeling

A homology model of NB2 (GIII.2) VP1 was built based on the Norwalk virus (GI.1, Fig 1B and 1C) capsid protein crystallographic structure (PDB: 1IHM)[3]. Chain B of 1IHM was chosen as a structural template for the target GIII.2 dimer sequence as it is the more complete, comprising residues 10–520 of the 530-residue GI.1 VP1. The Modeller 9.14 program [10,11] was used to generate 100 structural models of the GIII.2 monomer that were further evaluated based on their pseudo-energy ("DOPE") value [12] and the number of residues outlier on the Ramachandran plot [13] with the RAMPAGE webserver (http://mordred.bioc.cam.ac.uk/~rapper/rampage.php). We selected the structural model with lowest DOPE value (8 Ramachandran plot outliers) and generated the dimer by aligning two copies on both A and B chains of the GI.1 structure with Pymol [14]. The model was then neutralized with 34 Na+ ions, hydrated with TIP3P water molecules and minimized afterward with the steepest-descent method during 20000 steps while restraining the protein atoms and ions with harmonic potentials with a constant force of 1000 KJ/mol. We compared this GIII.2 dimer model to the GI.1 A-B dimer (both dimers with missing N- and C-termini modeled *ab initio*) by performing a 100 ns molecular dynamics simulation for both (see *Preparatory simulations for the N-terminal arms study* for details of the simulation protocols).

### Simulations

Molecular dynamics simulations were performed with the GROMACS simulation package version 5.0 [15] with the Amber 99SB-ILDN force-field [16] except for simulated annealing molecular dynamics, for which we used AMBER 9 [17–20]. Protocols are detailed in each subsection.

### Simulated annealing molecular dynamics

Simulated annealing molecular dynamics were performed in implicit solvent. The starting model was the GIII.2 dimer after homology modeling and minimization (see above). Residues 1 to 30 were set as flexible while residues 31 to 522 remained fixed. Under the protocol used, the system was heated at 1200°K for 1ps and stabilized for 2ps. During the 13 next ps we reached 300°K and stabilized the system at this temperature for 1ps. Finally we reached 0°K during 3ps and the coordinates of the trapped structure were written at the end of each cycle. This basic loop of 18 ps was iterated 199 times to explore the conformational space of feasible solutions for the N-terminal arm.

### Clustering

We developed in python a software to clusterize our trajectories [21]. We used the MDTRAJ package [22] for the trajectory reading and RMSD matrix calculation. Numpy 1.11 [23] was used for the hierarchical clustering. The Ward method [10] was chosen and its graphical classification of the trajectory was used to select the clustering level. The representative frame is defined by the structure with the lowest RMSD against all the frames which belong to the same cluster. The source code is available at https://github.com/tubiana/TTClust.

### Preparatory simulations for the N-terminal arms study

The starting structure was chosen as the representative structure of the main cluster for chain A obtained from simulated annealing molecular dynamics. We generated the 8 possible sets of protonation states for the 3 acidic amino acids Glu10, Glu22 and Asp152 (GIII.2 numbering). These structures were then neutralized with the appropriate number of Na+ ions (from 14 for the triply-protonated arm to 17 for the non-protonated arm) and solvated with TIP3P water [24] under periodic condition with a non-cubic box with a distance between the protein and the edge of the box of at least 1.3 nm. The structures obtained after energy minimization were used to initiate molecular dynamics simulations. An integration time step of 2 fs was used during the calculation and the neighbour list was updated every 25th step. Short range non bonded Van der Waals (Lennard-Jones) and Coulomb interactions were calculated within a cut-off radius of 1 nm. The long range electrostatic interactions were calculated with the particle mesh Ewald (PME) method [25] with a grid spacing of 0.16 nm. The long range Lennard-Jones interactions were analytically corrected for in the calculation of the pressure and the energy.

Water molecules were constrained using the SETTLE algorithm [26] and the covalent bonds in the proteins were constrained using the P-LINCS algorithm [27].

Two NVT equilibrations of 500 ps were performed, one at 150°K followed by another one at 300°K. The solvent and the protein were coupled separately to an external heat bath at 300 K with the velocity-rescaling thermostat [28] using a time constant of 0.1 ps.

Then we performed a NPT equilibration of 500ps. The pressure of the simulation box was kept at an average of 1 bar using the isotropic Parrinello-Rahman barostat [29] with a time constant of 2 ps and a compressibility of 4.5e^-5^ bar^-1^.

After the equilibration, the position restraints were removed. All other simulation parameters were the same as during the equilibration.

VMD 1.9.2 was used for RMSD calculations and Gromacs tool “rmsf” for the RMSF.

### Atomic model derived from the GII.10 cryo-EM map

An extended model was generated for the GI.1 crystallographic dimer (pdb ID: 1IHM, chains A and B). The cryo-EM map used is the available one for the GII.10 norovirus VLP to about10 Å resolution (EMDB ID: 5374)[8]. The interstitial regions (residues 220 to 230) of the crystallographic dimer were removed and the P dimer and S dimer were fitted separately with Chimera program version 1.10 [30]. The S dimer was first placed manually so that chain A was at its approximate position as one of the five A molecules around a fivefold axis. The S dimer was then adjusted by the auto-fit function of Chimera with a simulated map of 10 Å to finally reach a correlation coefficient of 0.89. The same protocol was used to fit the P dimer above the positioned S dimer, with a final correlation coefficient for the P dimer of 0.93. The S and P dimers were then connected by their two interstitial regions (residues 220 to 230) with Modeller.

### Simulations for the P/S domain interaction study

For this study, we used the same simulation protocol as for N-terminal arms study (see above) for replicas 1 and 2. The distance between the protein and the edge of the box was increased from 1.3 nm to 2 nm for replicas 3–5. We initially set a pre-production time of 5 ns before a production period of 100 ns but finally included the whole 105 ns as production.

The principal axis of the S domain was calculated with the Single Value Decomposition module of numpy [23]. This method is adapted from the UCSF Chimera 1.10 [30]. Only residues in beta sheets of the S domain (67–70, 79–84, 107–114, 122–128, 147–151, 158–162, 181–186, 202–210) were considered to minimize the noise due to the thermic fluctuation of the inter-strand loops. The height of domain S was calculated by aligning the P dimer axis on the Z axis. The center of mass of the jelly roll of the simulated protein was compared with the corresponding center of mass of the crystallographic GI.1 structure that was set to z = 0. Changing interactions between S and P domains were monitored with VMD [31] and plotted with matplotlib [32].

### Sequence conservation

29 norovirus capsid protein full sequences were retrieved, each one the representative sequence of a single genotype [6]. Genbank accession codes for GI members are: M87661 (GI.1, crystal structure), L07418, U04469, AB042808, AJ277614, AF093797, AJ277609, AF538679; GII members are U07611, X81879, U02030, X76716, AJ277607, AJ277620, AJ277608, AF195848 (GII.10, cryo-EM map), AY038599, AF427118, AB074893, AJ277618, AY113106, AY130761, AY130762, AY502010, AY502009; GIII members are AJ011099, AF320625 (GIII.2, TR-SAXS); GIV member is AF195847 and GV member is AY228235. An alignment of these 29 sequences was generated within Jalview software v2.7 [33] with the MUSCLE [34] webserver and default parameters. From this alignment, a sequence conservation score was computed at each position in Jalview. The score is based on the one used in AMAS [35] and in case of variation takes into account the number of conserved physico-chemical properties at a position. The conservation is scored from 0 (not conserved) to 11 (completely conserved).

A dendrogram for these 29 sequences was calculated on the phylogeny.fr webserver [36] with PhyML 3.1/3.0 [37] with the aLRT statistical test [38] and WAG substitution model [39]. The tree was then rendered with drawtree 3.66 [36].

### SAXS

#### VLP production, electron microscopy and dissociation

The capsid gene of norovirus GI.1 Norwalk virus (AY502016) was cloned into a baculovirus expression system as previously described [40]. The VLPs were harvested from Hi5 cells at five days post infection. The clarified supernatant was pelletized and applied to a 15–45% sucrose-PBS ultracentrifugation gradient (Beckmann SW40-Ti rotor) for 2 h at 4°C. VLP fractions were confirmed using EM and homogenous particles were pooled and concentrated to 2 mg/ml in PBS (pH 7.4). The VLP samples were applied to EM grids, washed once in distilled water, and then stained with 4% uranyl acetate. The samples were examined using EM (Zeiss EM 910). Prior to SAXS VLPs were dissociated into dimers through overnight dialysis at 4°C in CHES 0.01 M, pH 9. Protein concentration after dialysis was 1 mg/ml.

#### Data acquisition and analysis

Data were collected at the SWING beamline of Synchrotron Soleil (Saint-Aubin, France). Serial dilutions of the protein were measured (initial concentration, dilution 2 and dilution 4). The dialysis buffer was measured before and after each dilution. All buffer measurements were found to be superimposable and were averaged and subtracted from the protein solution measurements. Subsequent analyses were performed using various tools from the ATSAS suite, version 2.8.3 [41], as indicated. The three dilutions were analyzed in PRIMUS and found to be superimposable after scaling. We further analyzed the data from the undiluted sample. Experimental radius of gyration and Dmax were computed from the pair distribution function *p*(*r*) with GNOM from the AutoRg routine in PRIMUS, to be consistent with our previous analysis of the GIII.2 dimer data [9]. We also generated a real space envelope using an automated Python script that streamlines and standardizes the classical procedure of multiple envelope averaging. 30 envelopes were generated with DAMMIN and aligned with SUPCOMB. We finally took the average envelope and the lowest envelope corresponding to the envelope with all common points between all envelopes.

#### Rigid body modeling

We used EOM [42] for modeling with several conformations and DADIMODO [43] for modeling with a single conformation. To allow for sufficient flexibility we chose as flexible slightly enlarged versions of the termini and connecting segments. Thus in both cases the three rigid bodies were defined as residues 232 to 520 of both chains A and B (dimer of P), residues 29 to 217 of chain A and residues 29 to 217 of chain B. For DADIMODO that uses complete all-atom models, the N-terminus and C-terminus regions were added to the GI.1 crystallographic A-B dimer with Modeller 9.14. The DADIMODO genetic algorithm was used with the following parameters: At each step 20 offspring are generated by changing a random main chain dihedral angle in the flexible regions by a maximum of 30° and minimizing the resulting conformation. A population of 10 conformations is then selected by their fit to the SAXS data as computed with CRYSOL, with increasing selection pressure over a maximum of 300 generations.

### Structure images

Images and movies were rendered with Pymol 1.8.2.0 [14].

## Results

### Simulations on the GIII.2 VP1 show an asymmetrical behavior of the dimer and protonation-dependent conformations in the N-terminal arm

#### Homology modeling from the GI.1 VP1 dimer and simulations

**Fig 2:**
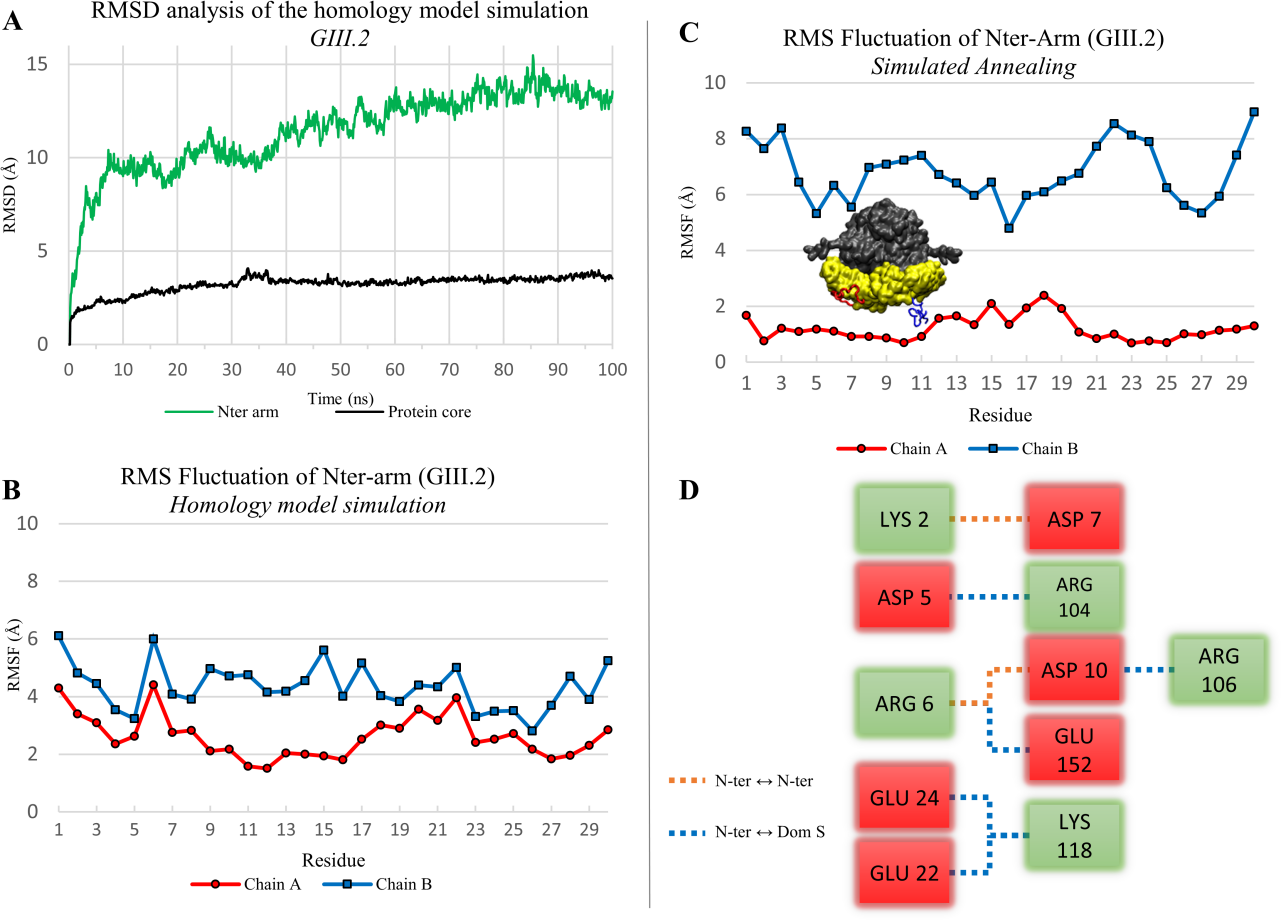
Dynamic properties and conformations of the N-terminal arm in a homology model of the GIII.2 dimer. (A) RMSD analysis of the homology model simulation (100 ns). The protein core (black curve) is defined by residues 31 to 512 (without N-terminal and C-terminal arms). The N-terminal arm is analysed separately (green curve). (B) RMS fluctuation of the N-terminal arms (residues 1 to 30) in the homology model simulation. Heavy atoms only were considered for the RMSD and RMSF calculation. (C) RMS fluctuation of N-terminal arms in the 199 endpoints of simulated annealing. A representative position of each arm is also displayed on the protein picture. (D) Salt bridge network found for the arm of chain A in 84% of the simulated annealing endpoints. Interactions within the arm are represented in orange and interactions with S domain in blue. The color of the box is defined by the charge of the amino acid (negative in red, positive in green).

In the absence of a crystal structure of the GIII.2 VP1 of which we experimentally characterized the self-assembly behavior, both statically [44] and dynamically by TR-SAXS [9], we generated a homology model of the GIII.2 VP1 dimer from the GI.1 VLP crystal structure. The amino acid sequence of chain B is more complete in the crystal structure, with residues 10–520 (out of 530) present in the model, corresponding to residues 10–511 (out of 522) of the GIII.2 VP1. We therefore used chain B as a template for both molecules A and B of the GIII.2 model, adding the missing 9 N-terminal and 11 C-terminal residues in the modeling. We performed a 100 ns MD simulation to assess stability of this model. S and P domains maintained both their internal fold and their initial interactions P to P and S to S across the two-fold axis. RMSD of non-hydrogen atoms for this VP1 dimer core, excluding N and C-termini, plateaued to less than 4 Å (Fig 2A, black curve). The C-termini were dynamically flexible (not shown), as expected from parts not seen in any of the three crystallographic molecules. The N-terminal arms strongly diverged from the starting structure (Fig 2A, green curve), including residues 10–30, the counterparts of which are fully ordered in crystallographic molecule B. A simulation of the GI.1 crystal structure A-B dimer with termini added *ab initio* that we performed in parallel behaved very similarly, plateauing slightly below 3 Å RMSD for core P and S but with N-termini diverging (not shown).

We previously noted that in the GI.1 crystal structure ordering of the N-terminal arm from residue 10 in chain B (while it is disordered up to residue 29 in chains A and C [3]) involves clustering of three acidic amino-acids that seem conserved in GIII.2 VP1 and whose charges do not seem compensated by nearby basic residues [44]. Indeed, the initial disordering of the N-terminal arms in the simulations seems linked to initial electrostatic repulsion between negatively charged amino acids among this cluster (D13-E24-D156 for GI.1 and D10-E22-E152 for GIII.2). Furthermore, the N-terminal arm of chain B displayed higher mobility than that of chain A (Fig 2B).

#### Simulated annealing finds an alternate and stable N-terminal arm conformation

In view of this, we sought to generate in a GIII.2 VP1 dimer alternate conformations of the N-terminal arms remote from their initial, unstable conformation. Our goal was to obtain starting points from which to simulate various alternate charge states of the arms. To this end we performed simulated annealing on our minimized homology model (initially perfectly symmetrical), keeping residues 31–522 fixed (S1 Movie).

We used our homemade trajectory clustering tool [21] to analyze the conformations of the N-terminal arms during the 199 simulated annealing iterations. Using the same cut-off on hierarchical clustering on both chains we find a larger number of distinct clusters on chain B than on chain A (S1 Fig). For the B chain’s N-terminus, a cut-off of 8 arbitrary units yields 7 clusters (4 mains) with an average RMSD between the members of the clusters (spread) of 8.2 Å (Table 1). In contrast, for chain A only three clusters are present at this hierarchical distance and a preferred conformation emerges. There are two clusters with a spread of 0.5 Å and 1.0 Å constituting 83.9% and 14.5% respectively of all the chain A N-terminal conformations. The single sharp difference between the two clusters is a loop between residues 11 to 19 that occupies alternate positions in either cluster. Accordingly, RMSF along the N-terminal arm is low for chain A in the simulated annealing, with a single bump for this loop, while it is high for chain B (Fig 2C).

**Table 1:** Simulated annealing clustering results. For each cluster the size is the number of endpoints and the spread the average RMSD between all endpoints composing this cluster. The spread can also be represented by the compactness of nodes in a dendrogram (see S1 Fig). a, the three members of this cluster are the first three endpoints.

CLUSTER	SIZE	SPREAD (Å)
**Chain A**		
1	3^a^	5.3
2	29	1
3	167	0.5
**Chain B**		
1	3	6.8
2	14	10.1
3	12	8.2
4	52	8.5
5	35	8.2
6	43	8.1
7	40	7.7

On examination of the endpoints, the N-terminal arm makes few interactions with its S domain for chain B. In contrast, in chain A there are numerous salt bridges between the N-ter arm and the S domain (Fig 2D). Particularly, D10, E22 and E152 pair with R106 (corresponding to GI.1 R110), K118 (GI.1 K122) and R6 (GI.1 K6), respectively.

In summary, assuming basal protonation states and thus all negative charges of the D10-E22-E152 triad, not only is their clustering initially unstable, but the N-terminal arm can find a stable conformation (possibly a local minimum) where the three residues contribute three anchor points through salt bridges with the S domain.

#### Simulations reveal a protonation-dependent propensity to come back to the GI.1 arm conformation

**Fig 3:**
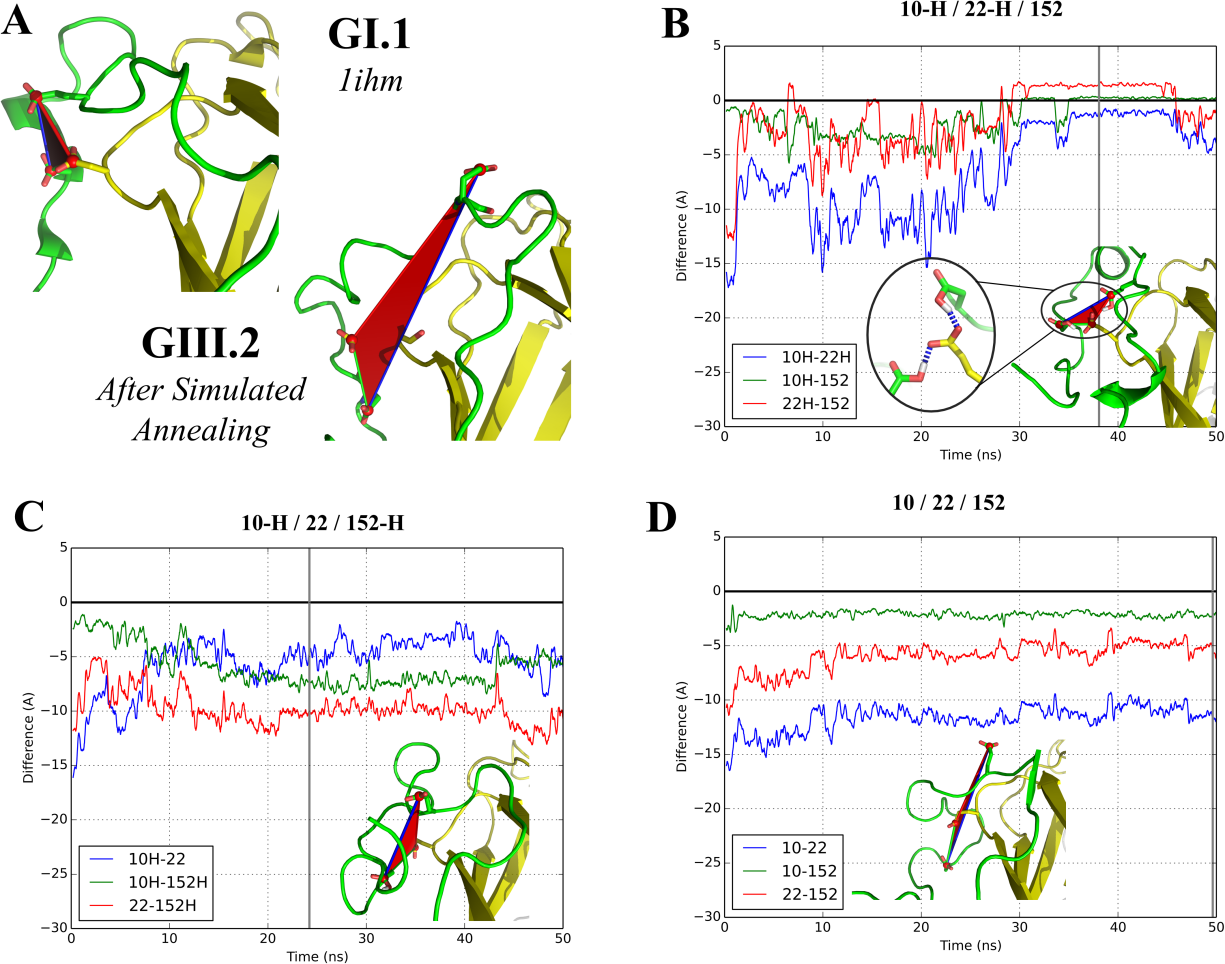
Molecular dynamics simulations in different sets of protonation for the three acidic residues clustered in the GI.1 VP1. (A) Comparison of N-terminal arms in the GI.1 crystal structure (chain B) and after simulated annealing of our GIII.2 model. Color code as in Fig 1A. The side chains of the three acidic residues considered are displayed. (B-D) Evolution of the difference distances between D10, E22 and E152. A value of 0 corresponds to restoration of the initial distance in the GI.1 crystal structure while a negative value indicates a larger distance (see text for details). The illustrations represent the geometry of a representative frame of the largest cluster of each trajectory (S2 Fig). The gray vertical line indicates the frame from which the picture originates.

Since we found a favored conformation of the fully deprotonated arm by simulated annealing (the one accounting for 84% of endpoints in chain A, see above), we next assessed the protonation dependence of the N-terminal arm conformation and mobility by starting from this conformation and varying charge states of the arm. The arm makes only intramolecular contacts in both the crystallographic and simulated annealing conformations. Therefore for this experiment only we worked with a monomer. We generated the eight possible protonation states of the triad, from D10 / E22 / E152 (all three residues deprotonated, as in the above analyses) to D10-H / E22-H / E152-H (all three residues protonated). We ran a 50-ns simulation for each of these eight combinations. Particularly we monitored the three difference distances between each pair of side chain carboxyls compared to the corresponding pair in the GI.1 crystal structure, for instance for D10 and E22 of GIII.2 (corresponding to D13 and E24 of GI.1) we considered

d(D13,E24)−d(D10,E22)

where d(x, y) is the distance between the carbon of the side chain carboxyl of residue x and that of residue y (Fig 3A).

In the case where all three residues are deprotonated (Fig 3D, S2 Movie), we find that the arm conformation relaxes into a position that remains close to the simulated annealing position. This is evidenced by the representative frame that is found at the end of the simulation and by the two difference distances that remain stable at high negative values, as E22 remains far from the other two residues of the triad. Overall the pattern of salt bridges depicted in Fig 2D remains in place. Indeed D10 and E152 draw slightly closer because they simultaneously interact with R6. The same behavior is found when D10 and only D10 is protonated (**S3**B Fig). In contrast, large changes occur as soon as either E22 or E152 are protonated. Two cases are then found. When either D10 or E22 remains deprotonated (Fig 3C, S2 Movie and S3B, S3C, S3D and S3E Fig), the N-terminal arm becomes dynamically disordered. As interactions between protonated residues and basic residues (formerly salt bridges) break, the corresponding acidic residues drift away. The difference distances fluctuate along the simulations and generally remain below -4 Å, indicating a lack of interaction between the acidic residues. However, when both D10 and E22 are protonated (Fig 3B, S2 Movie and S3F Fig), the initial fluctuation leads to grouping of the D10-E22-E152 triad into a stable cluster as in the GI.1 crystal structure.

These results suggest this clustering is likely a general property of the norovirus VP1 but also indicate that two protonation events are necessary in order for the N-terminal arm to fold into its final state in chain B. At the neutral pH at which the simulations were performed, this double event is unlikely without modifications of the chemical environment. In the discussion section below we discuss possible chemical clues that could be involved during capsid assembly.

### Simulations on an extended form of the GI.1 dimer reveal a propensity for alternate, asymmetrical S-P interactions

#### Simulations starting from an extended dimer model derived from cryo-EM data of the GII.10 VLP

**Fig 4:**
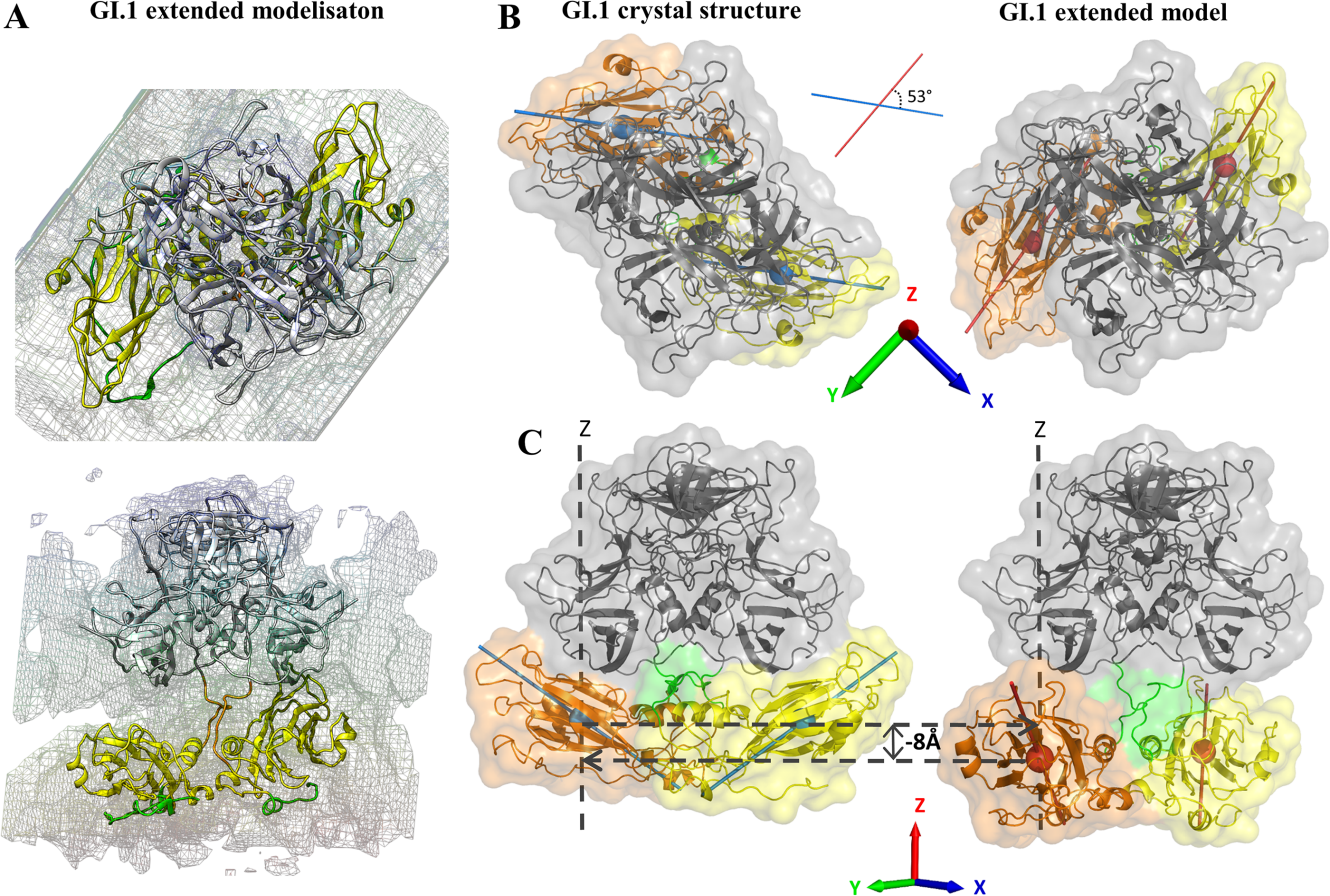
Construction of an extended form of GI.1 VP1 dimer based on the available cryo-EM data. (A) Fitting of GI.1 A-B dimer in the GII.10 cryo-EM Map (EMDB: 5374 [8]). The connecting segments were generated with modeller 9.14. The termini were not modeled. Color code as in Fig 1A. (B) Definition of angle and height of S domains relative to the crystal structure's. The reference is the dimer of P, the height is of the center of mass of S along the P dimer axis (*O*Z) and the angle is between the S axes as defined in the methods section. In this panel, S of chain A is in orange and of chain B in yelllow.

The compact form of VP1 with P domain interacting with S domain (Fig 1A) has been found so far only with the GI.1 VLP crystal structure. Our simulations show that this form is stable in the isolated dimer at the 100 ns timescale for both GI.1 VP1 and a homology model of GIII.2 VP1 (Fig 2A). However, we previously found by SAXS that the GIII.2 dimer has a larger radius of gyration than the GI.1 crystallographic dimer and that the GIII.2 VLP has a larger diameter than the GI.1 VLP [44]. Both GV virion and GII VLP have been imaged by EM as a double shell, with a cage of P dimers raised off the S shell and no contact between S and P [7][8]. We thus wanted to assess whether the GI.1 dissociated dimer could assume a similarly extended conformation. We took advantage of the availability of the EM map for GII.10 VLP to generate a starting extended GI.1 dimer model by successively fitting a GI.1 S dimer then a P dimer into this map (Fig 4A). As in previous reports we find the P dimer and S dimer rotated and separated compared to the crystal structure [7][8]. If we take the P dimer as a reference, the axes of the S domains are at an angle of some 53° (Fig 4B) and their center of masses are some 8 Å below a plane orthogonal to the dimer axis (XY plane in Fig 4C).

**Fig 5:**
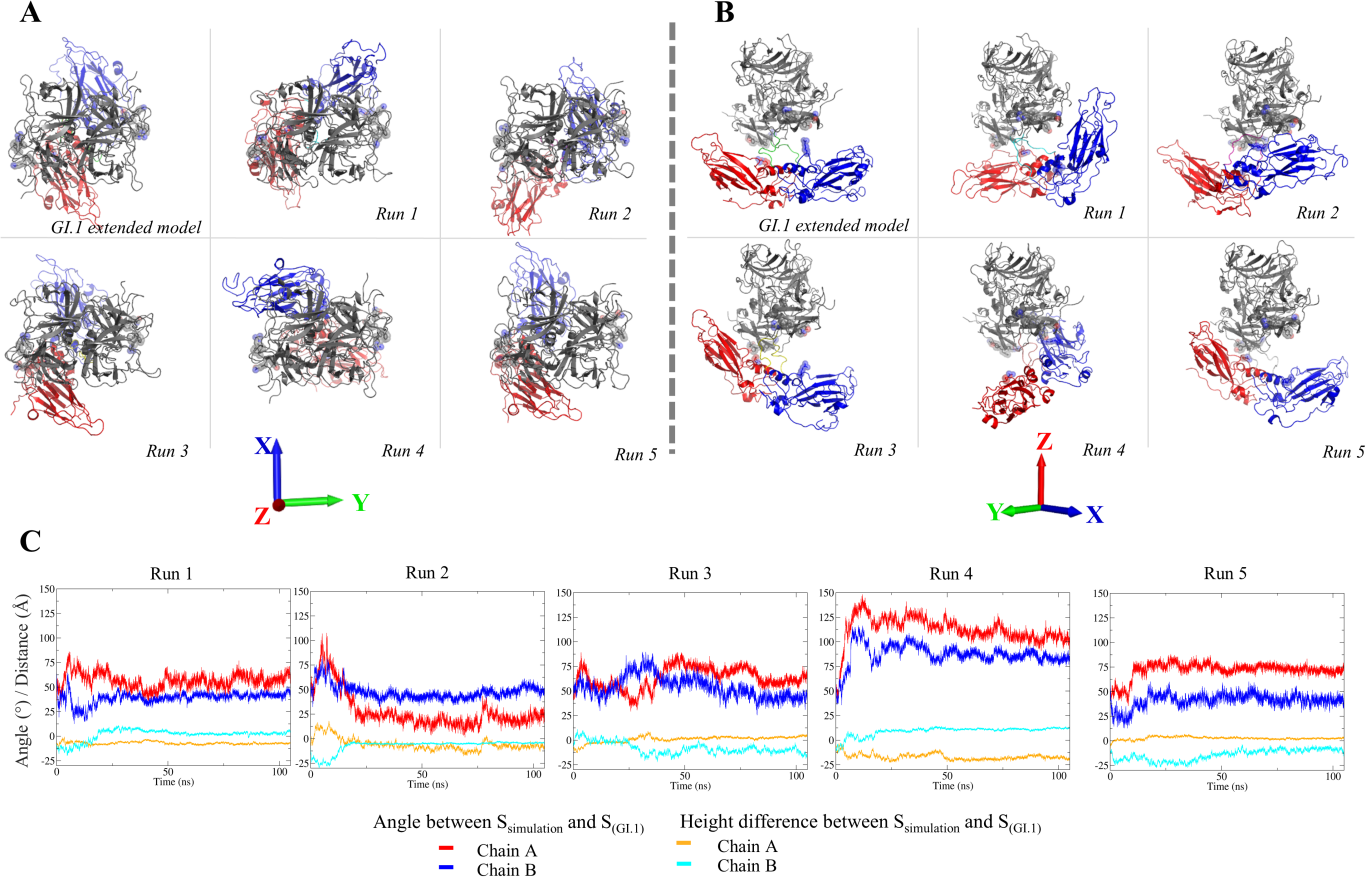
Simulations of the extended GI.1 dimer. Top (A) and front (B) view for the extended model of Fig 4 and the endpoints of 5 simulations. S domains are red for chain A and blue for chain B. (C) Evolution of the angles and heights of S domains as defined in Fig 4. Values of 0 correspond to the S conformation of the GI.1 crystal structure.

We performed five 105 ns distinct simulations starting from this extended model (this time without the termini modeled in) to investigate the stability of the dimer in this conformation (S3 Movie). We monitored the angles and heights of S domains with reference to the crystal structure A-B dimer and examined the endpoints of the simulations (Fig 5). All five simulations quickly diverge from the initial extended model and its initial values of 53° and -8 Å (see for instance Fig 5A and the angles in Fig 5C). There is a common behavior in all five simulations in that in all of them an S domain quickly comes into contact with a P domain and stays attached to it (Fig 5B), as evidenced by stabilisation of its relative height (Fig 5C).

Furthermore, the contact between S domains, that is found in both crystal dimers, is also maintained throughout the five simulations. However, no two simulations are the same and none comes back to a conformation similar to the crystallographic A-B dimer that we keep as a reference. The first simulation comes closest and indeed it is the only one in which both domains S come into contact with their P domains. In the other four simulations one S domain (either of chain A or of chain B) comes into contact with a P domain at an early stage and subsequently remains in interaction with it. Simulation 4 is an outlier in that the interaction is across molecules, between domain S of chain A and domain P of chain B. In all other simulations the interaction is within molecules as in the crystal structure, either within chain A (simulations 3 and 5), within chain B (simulation 2), or within both (simulation 1).

#### S-P interaction sites and their sequence conservation

**Fig 6:**
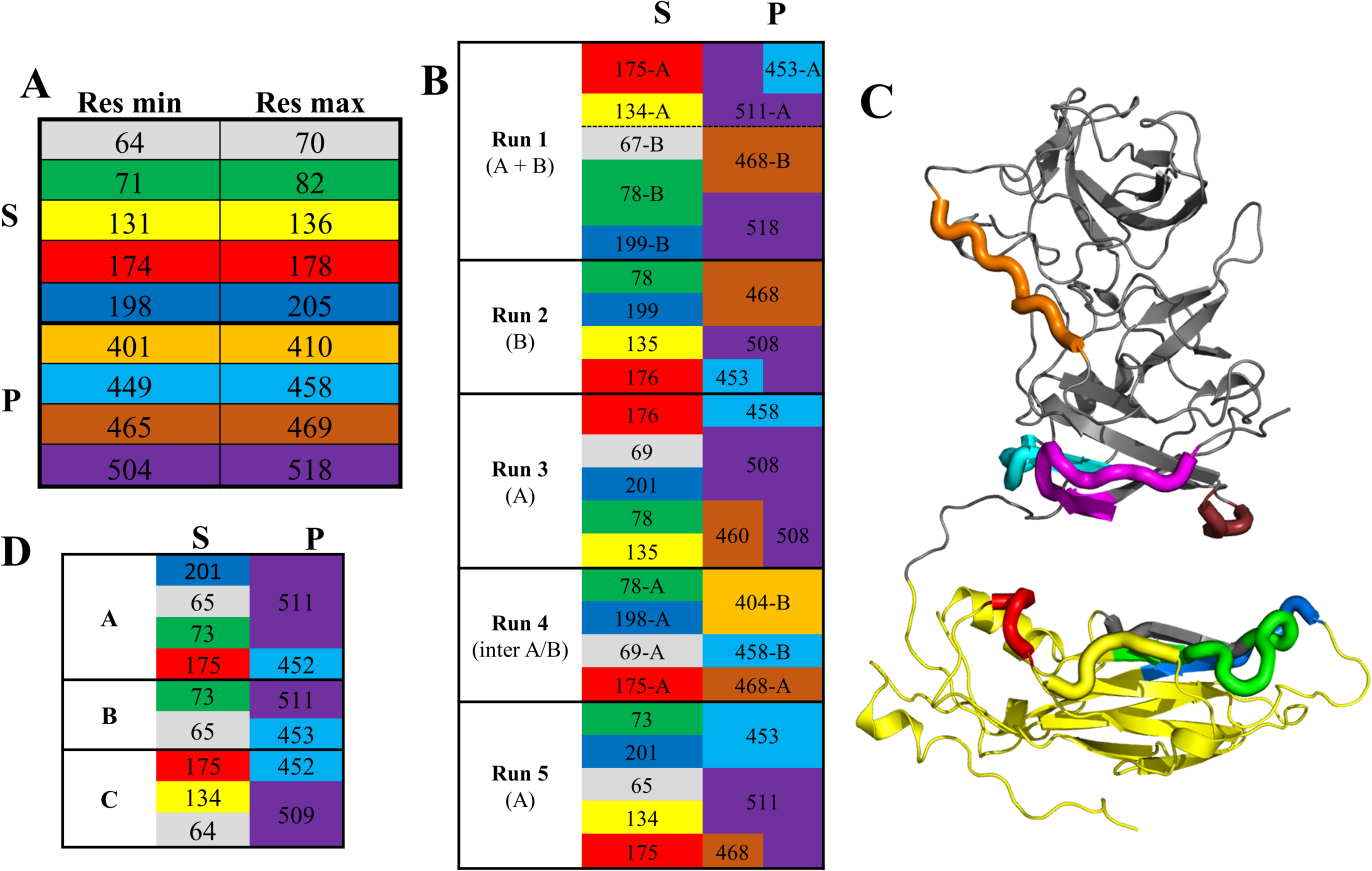
Segments of S and P domains interacting with one another. (A) Limits of amino acid segments found in S (top) interacting with P or in P (bottom) interacting with S in any of the 5 simulations of Fig 5. Each segment is associated with a color kept in the rest of the figure. (B) Interactions between segments in all simulations. Interacting segments between S (left) and P (right) are presented on the same line. The segment's residue indicated is the one with the strongest and/or most frequent interaction (S4 Fig). Chains are indicated as needed (run 1, where both chains have S-P interactions; run 4, where cross-chain interactions are observed). (C) Location of the segments on the (extended) GI.1 VP1 structure. (D) Same table as (B), but for the three crystallographic molecules.

We next looked at the residues involved in the interactions between the S and P domains along all five simulations (S4 Fig) and compared them to the GI.1 VLP A, B and C molecules. In the simulations, after stabilisation of the S-P contacts, we find overall five segments in S interacting with four segments in P (Fig 6A). If we set aside the outlying simulation 4, in which the interaction is intermolecular and involves a contact segment (around residue 404) not found in other simulations, a consistent pattern emerges (Fig 6B and 6C). There are two interacting regions at the top of domain S made of segments 174–178 and 131–136 on the one hand and of a continuous patch made of neighboring segments 64–70, 71–82 and 198–205 on the other hand. These two regions are contacted by three loops at the bottom of domain P, a long central segment 504–518 and its flanking loops 449–458 and 465–469. Depending on the relative orientation of S and P, 504–518 may contact either or both S regions.

**Fig 7:**
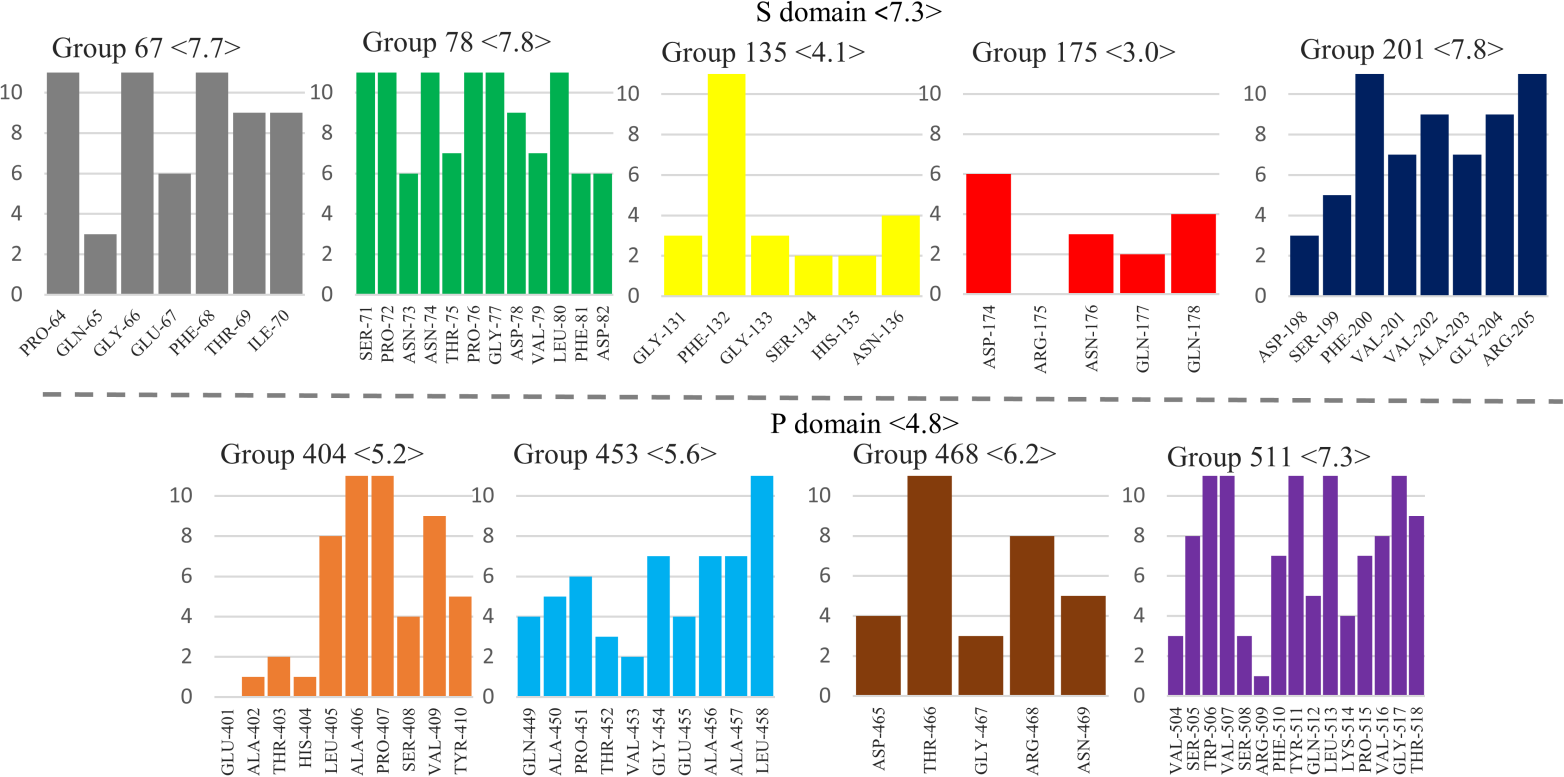
Sequence conservation among noroviruses of the S-P interaction segments. The color code is the same as Fig 6. The conservation score was calculated with Jalview [33] on a scale from 0 to 11 based on one representative VP1 sequence for each of 29 genotypes [6]. Averages for segments and domains are indicated in brackets.

Despite different orientations of the domains, a similar pattern is found also in the crystallographic molecules A, B and C (Fig 6D). The segments involved are the same, and alternate interactions of loop 504–518 reflect the small conformational differences in the three quasi-equivalent molecules. The only exception is the short loop 465–469, belonging to P domain, that is involved in S contacts in all simulations but does not contact the S domain in any of the three crystallographic molecules. Instead in the VLP it caps segments 71–82 and 131–136 of one molecule in a neighboring dimer. Segment 401–410 is also involved in interactions (between P domains) in the VLP. Thus, all the P-S interacting segments that are found in our GI.1 dimer simulations participate in key interactions in the GI.1 VLP crystal structure, and all but one are the elements that position domain P relative to domain S. We examined their sequence conservation in the five major genogroups of noroviruses (Fig 7).

Most contain several very conserved residues, above 8 on the discrete scale (where 11 is strictly conserved and 10 very conserved with physico-chemical properties strictly conserved), while the average score for VP1's entire sequence is 5.84. This higher conservation is particularly noteworthy for the loops in domain P since conservation is lower in this domain (average score of 4.8). However, the first domain S interacting region (segments 131–136 and 174–178) is surprisingly nonconserved. In particular the polar segment 174–178, involved in the interaction with P and/or the interstitial region, displays very low conservation. For instance, R175-N176 is an amino acid pair that is most often prominently involved in P/S interactions in our simulations (S3 Fig) and in two of the three crystallographic molecules (Fig 6D). Yet in our sample of 29 norovirus genotypes it is present only in GI.1 and GII.10.

#### New SAXS data reveal a very extended and asymmetric GI.1 VP1 dimer

To further probe the conservation and differences between dimers of different genogroups, we acquired SAXS data on the GI.1 dimer dissociated from purified VLP by high pH and low ionic strength, as we did previously for the GIII.2 dimer [9]. We could use a sample setup more suited to a 115-kDa protein than in our previous GIII.2 work, yielding more accurate data on a more appropriate range of wavenumbers (0.01 Å^-1^ < q < 0.42 Å^-1^). In their common range the dimer data are comparable between GI.1 and GIII.2 (S5 Fig) and their analysis in terms of distance distributions yield similar radii of gyration of 42 Å and 43 Å, respectively, for a maximum dimension of 150 Å. This shows that the two dimers are comparable in their extension. The derived SAXS molecular envelopes are also comparable for GI.1 and GIII.2 as cylinders with a central bulge (not shown). However, the radii of gyration and maximum dimensions are much higher than computed from the X-ray crystallography or cryo-EM derived experimental model (Table 2). In all our simulations from the cryo-EM model (described above), radii of gyration also keep well below 42 Å, settling at or near the cryo-EM value (S6 Fig).

**Table 2:** Radii of gyration and maximum dimensions of crystallographic dimer, cryo-EM based model, SAXS data and Dadimodo model. Radii of gyration and Dmax were calculated with Crysol for structural models, for which termini were included. P(R) analysis was used for experimental data.

	Radii of gyration (Å)	Dmax (Å)
**SAXS GI.1**	43.3	150
**SAXS GIII.2**	42.0	150
**GI.I Xray**	32.7	99.6
**Cryo-EM Model**	35.1	110.9
**Dadimodo**	43.7	202.1

**Fig 8:**
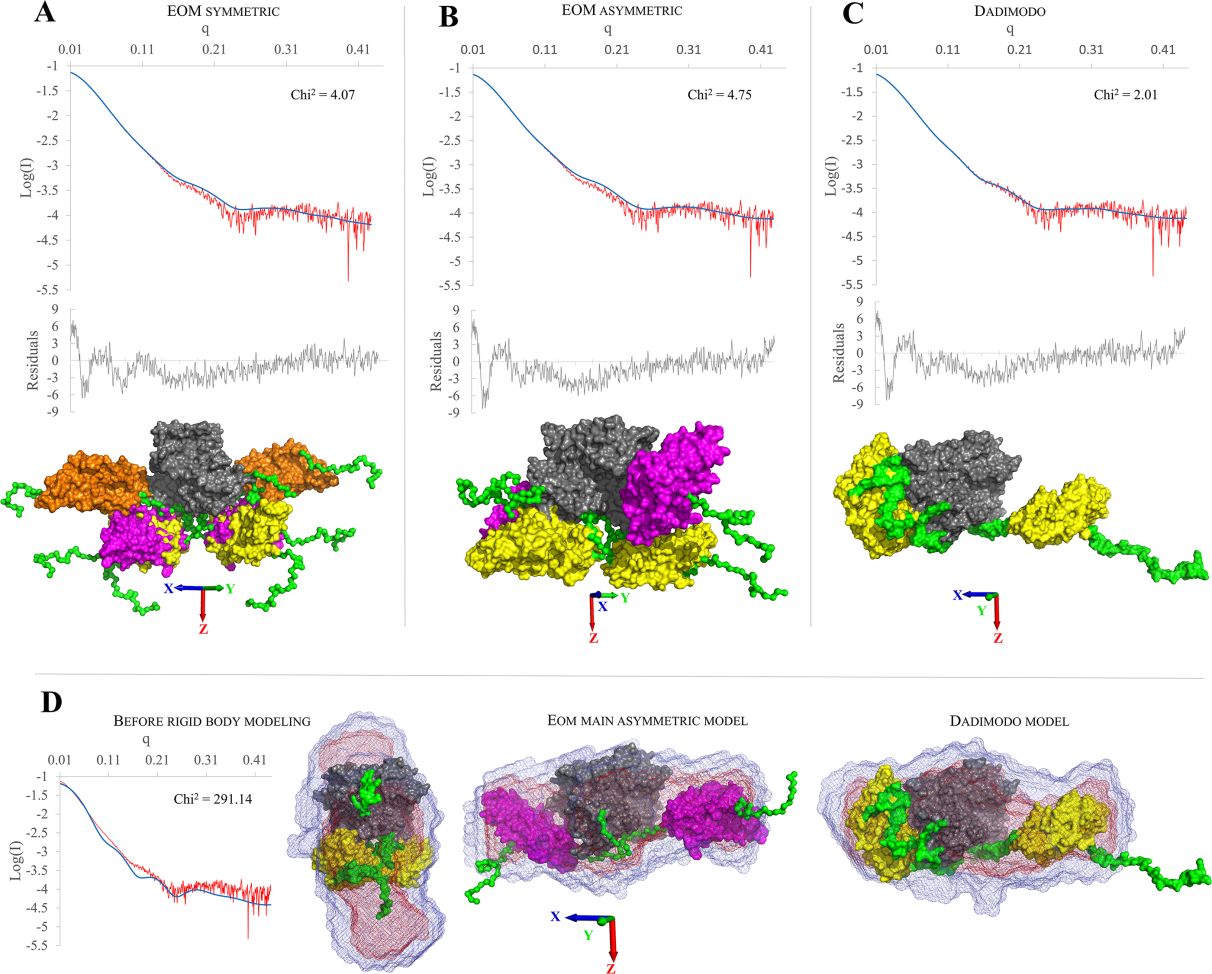
Solution structures of dissociated GI.1 dimer obtained by rigid body modeling of SAXS data. The fit of the curve computed from the model (in blue) to the experimental data (in red) and their associated reduced residuals (in gray) are displayed atop the models. (A) EOM results for 10000 symmetric models. The population of 3 selected models is displayed superposed on their P domains (in gray) with the 3 pairs of S domains in yellow, orange and magenta. (B) EOM results for 10000 asymmetric models. The population of 2 selected models is displayed superposed on their P domains (in gray) with the 2 pairs of S domains in yellow and magenta. (C) Fit of the single Dadimodo model. (D) Comparison of the GI.1 dimer before rigid body modeling (1IHM with arms modeled in, the fit to the experimental data is also shown) with the two main models from EOM (asymmetric) and Dadimodo. The best superposition of the *ab initio* envelope computed from the SAXS data is displayed for each model.

The limitations of the GIII.2 data did not allow a more precise definition of the solution dimer form, as they were consistent with several qualitatively different associations of domains (not shown). The higher quality of the present GI.1 data (S5 Fig) allows more discriminating reconstruction of the solution structure by rigid body modeling. We set the N- and C-termini and the two connecting segments as flexible while keeping two S domains and one P dimer as rigid (see material and methods). We either used the strategy of generating 10000 random conformers and selecting a subset best matching the data [42], or the strategy of single-molecule modeling by perturbation from a starting conformation then selection based on fit to the data of intermediate models [45][43]. The former strategy was less successful than the latter in getting a good fit to the data, either when constraining models to two-fold symmetry (Fig 8A) or when generating non-symmetrical models (Fig 8B).

These searches result in a selection of 3 symmetric models whose combination yields a χ^2^ goodness of fit of 4 or of 2 asymmetric ones with a χ^2^ near 5, respectively (Table 3). In contrast, we obtain single models that fit our data better (χ^2^ in the range 2–2.5) by using the genetic algorithm implemented in the DADIMODO software, where generation of populations of random conformational variants is cycled with selection on their fit to the data [43]. The model with the best fit is shown on (Fig 8C). In all cases, we obtain models with the S domains separated and coming on either side of the P dimer, either as major components of multi-model ensembles (Fig 8A and 8B, Table 3) or as the single-model solution (Fig 8C). When not constrained to two-fold symmetry solution (Fig 8B and 8C) the solutions also display a pronounced asymmetry with one S close to the P dimer while the other extends away from it, a shape that matches well the molecular envelope while the crystallographic dimer does not (Fig 8D).

**Table 3:** Individual χ^2^ for EOM models, GI.1 crystallographic dimer and Dadimodo model. EOM models are presented with their fraction used to fit the experimental data. χ^2^ are calculated with Crysol [46]. a, for this model only the program FOXS [47] is used for computing χ^2^. The RMSD was calculated with the VMD visualizer tools using the Dadimodo model as reference. All proteins were aligned on the P domain and the RMSD was calculated based on the S/P domains position.

**Symmectric models**			
**Model**	**Used for (%)**	**χ**^**2**^	**RMSD (Å)**
1191	29	30.455	45.2
3878	57	13.51	60.7
8091	14	35.53	33.4
combination		4.07	
**Asymmetric models**			
1590	27	24.4	38.2
3350	73	7.1	12.2
combination		4.75	
**Other models**			
GI.1 xray		326^a^	31.6
Dadimodo		2.01	0

## Discussion

The norovirus protein VP1 forms stable dimers of which 90 self-assemble to form the major protein part of the capsid. VP1 is a two-domain protein harboring two points of flexibility, as seen in the crystal structure of the GI.1 VLP (Fig 1A). The first point of flexibility is a *ca* 10-residue segment between domains S and P that allows S dimers to assume either a "bent" conformation (dimer A-B) or a "flat" conformation (dimer C-C) [3]. The classical quasi-equivalence in the norovirus capsid allows environments of all molecules to be almost identical and thus A, B and C molecules display very limited adjustments in GI.1 VLP. From this observation Prasad et al [3] drew the most simple model of norovirus capsid self-assembly: A-B and C-C dimers would interconvert in solution, the proper conformation being incorporated at each point into a growing capsid. Indeed, our 100-ns simulations of a GI.1 crystallographic dimer (not shown) or of the GIII.2 model based on it (Fig 2A) would be consistent with this view of limited conformational range. However, we showed by SAXS that for the GIII VP1, compared to the GI.1 crystal structure, a more expanded VLP assembles from more extended dimers [44][9]. Here we show by SAXS that similarly dissociated GI.1 VP1 dimers assume as extended a conformation (S5 Fig). This shows that for GI.1 too the available range of conformations of VP1 goes well beyond the slight differences between A-B and C-C dimers in the GI.1 VLP. Indeed, rigid body modeling shows that the solution structure of the dimer is not simply a separation of P dimer and S dimer as for instance in the GII VLP [8], combined to extension of the mobile termini (Table 2). The solution structure is actually far out of the range of conformations assumed by VP1 in assembled capsids. A major difference is a prominent break of two-fold symmetry with one S domain in contact with the P dimer while the other projects away from it (Fig 8). Simulations starting from an extended GI.1 dimer based on the available GII.10 cryo-EM map (Fig 4) also feature asymmetric conformations, most of the time with only one S interacting with P (Fig 5,S3 Movie). The interactions involve the same segments of S and P as in the GI.1 crystal structure, but some of the contacts are not found in the final capsids (Fig 6). This further establishes the capability of the GI.1 VP1 dimer to assume conformations not seen in the crystal structure.

These results raise the question of the possible status of strongly asymmetric dimers in norovirus capsid assembly. From the large contact area between the five S domains around the five-fold axis, Prasad et al surmised that the initial assembly nucleus would be a pentamer of dimers and naturally considered that this nucleus would grow symmetrically [3]. Thus the next step would be a decamer of dimers with five-fold symmetry preserved, continuing the growth of a spherical piece of capsid. With pronouncedly asymmetrical dimers, the first step is not compromised (indeed the extension of one S domain away from the rest of the dimer may facilitate formation of an initial S-based pentamer of dimers), but symmetrical capture of five further dimers becomes problematic. Indeed, we found that the major intermediate in GIII.2 capsid self-assembly, although a 10- or 11-mer of dimers, is not a spherical piece of capsid. Instead it is very elongated and consistent with two connected pentamers of dimers [9]. The tendency for symmetry break of VP1 dimers we find here gives a possible structural basis for that unexpected observation.

Subsequent formation of the quasi-sixfold axes that feature in the architecture of the T = 3 norovirus capsid relies on the ability of the S domain to form also hexamers with almost the same pairwise contacts as in S pentamers. Interestingly, differences with the pentamers involve the second point of flexibility in VP1: The N-terminal arm that is disordered up to residue 29 in molecules A and C in the GI.1 VLP. Its ordering from residue 10 in molecule B actually cements the S hexamer by setting up on the one hand interactions between arms around the quasi-sixfold axis and on the other hand interactions with the nearby domains S of C molecules. However we show here that this arm conformation is unstable as is (Fig 2) due to a cluster of acidic amino-acids, two in the switching part of the arm and one on the surface of the S domain (Fig 3). For the GIII.2 dimer, we even find an alternate arm position that is highly stable (S1 Movie) due to salt bridges with domain S. Interestingly, we come back to clustering of the three acidic residues if and only if we protonate the two arm residues (Fig 3, S2 Movie, S3 Fig). Our findings thus highlight a switch in the VP1 N-terminal arm that requires neutralization of two of its negative charges to assume the conformation seen around quasi-sixfold axes. Such a mechanism requires time and/or the right triggers for formation of this part of the capsid. This would explain our biophysical observation that there is a slow step in the self-assembly of VP1 VLP going from the aforementioned (and presumably pentamer-based) intermediate to the full capsid [9]. In that previous work we triggered self-assembly by a salinity and pH jump from 9 to 6, so protonation of the two acidic residues still requires a shift in their pKa brought by a changing environment during assembly. In the GI.1 VLP crystal structure, protonation and stabilization of the B molecule N-terminal arm may have been favored by the crystallization pH of 4.8 [3]. But during assembly, that presumably occurs in a non-acidic cellular compartment, two alternate possibilities may be more relevant. First, ions may intervene to neutralize the charges of the two arm residues. Second, these charges may also be neutralized by the small basic norovirus protein VP2. VP2 is present in infectious norovirus particles in a small number of copies and is thought to mediate genome packaging during assembly [48]. Its involvement also in neutralization of the N-terminal arm is a particularly attractive speculation as it would provide a simple way to link capsid completion to genome encapsidation.

In conclusion, we find that the dissociated norovirus VP1 dimer tends to assume asymmetric conformations in solution through the two flexible points in its two-domain structure. We propose that this feature is functionally important in the assembly process.

## Supporting information

S1 Fig **Clustering of N-terminal arm simulated annealing endpoints (A) for chain A and (B) for chain B**. Top, clustering dendograms. The cutoff used to generate clusters from hierarchical classification is represented by a gray bar (8 for both chains). Bottom, each successive frame is assigned its cluster color. Graphics were generated with the TrajectoryClustering program (available at https://github.com/tubiana/TrajectoryClustering).
(TIF)

S2 Fig Clustering of N-terminal arm conformations along simulations with different protonation states. For every cluster, the size (number of frames in the cluster) and the spread (average rmsd between all cluster’s frames) are given. The number of the cluster depends on its order of appearance in the trajectory. A color code was attributed to each cluster.
(TIF)

S3 Fig Molecular dynamics simulations in different sets of protonation for the three acidic residues clustered in the GI.1 VP1. As for Fig 3, (A-F) Evolution of the difference distances between D10, E22 and E152. A value of 0 corresponds to restoration of the initial distance in the GI.1 structure while a negative value indicates a larger distance (see text for details). The illustrations represent the geometry of a representative frame according to the clusterisation of each trajectory (S2 Fig). The gray vertical line indicates the frame from which the picture originates.
(TIF)

S4 Fig Timelines of S domain interactions with P domain for simulations from an extended form of the GI.1 dimer. Residues inside a rectangle with the same colour belong to a single segment as defined in Fig 6. Interactions were sampled every 100 ps with VMD [31] and graphics made with matplotlib [32].
(TIF)

S5 Fig Comparison of SAXS results of the GI.1 (blue) and GIII.2 (orange) VP1 dimer. (TIF)

S6 Fig Timelines of radii of gyration during simulations from an extended form of the GI.1 dimer. Values for the crystallographic model and our cryo-EM derived model (without termini) are indicated.
(TIF)

S1 Movie Simulated annealing trajectory. Representation as in Fig 2C, with the N-terminal arms of chains A and B in green and emerald, respectively. Rendered with VMD.
(MP4)

S2 Movie 50 ns molecular dynamics simulations in three different sets of protonation for the three acidic residues clustered in the GI.1 VP1. The protonation states and representation are the same as for Fig 3.
(MP4)

S3 Movie 105 ns simulations of the extended GI.1 dimer. The 5 replicas are displayed as in Fig 4.
(MP4)
