# Supplementary figures and images for: Dynamics and asymmetry in the dimer of the norovirus major capsid protein
Source: PLoS One. 2017 Jul 27;12(7):e0182056. doi: 10.1371/journal.pone.0182056 (PMC5531542; doi:10.1371/journal.pone.0182056)

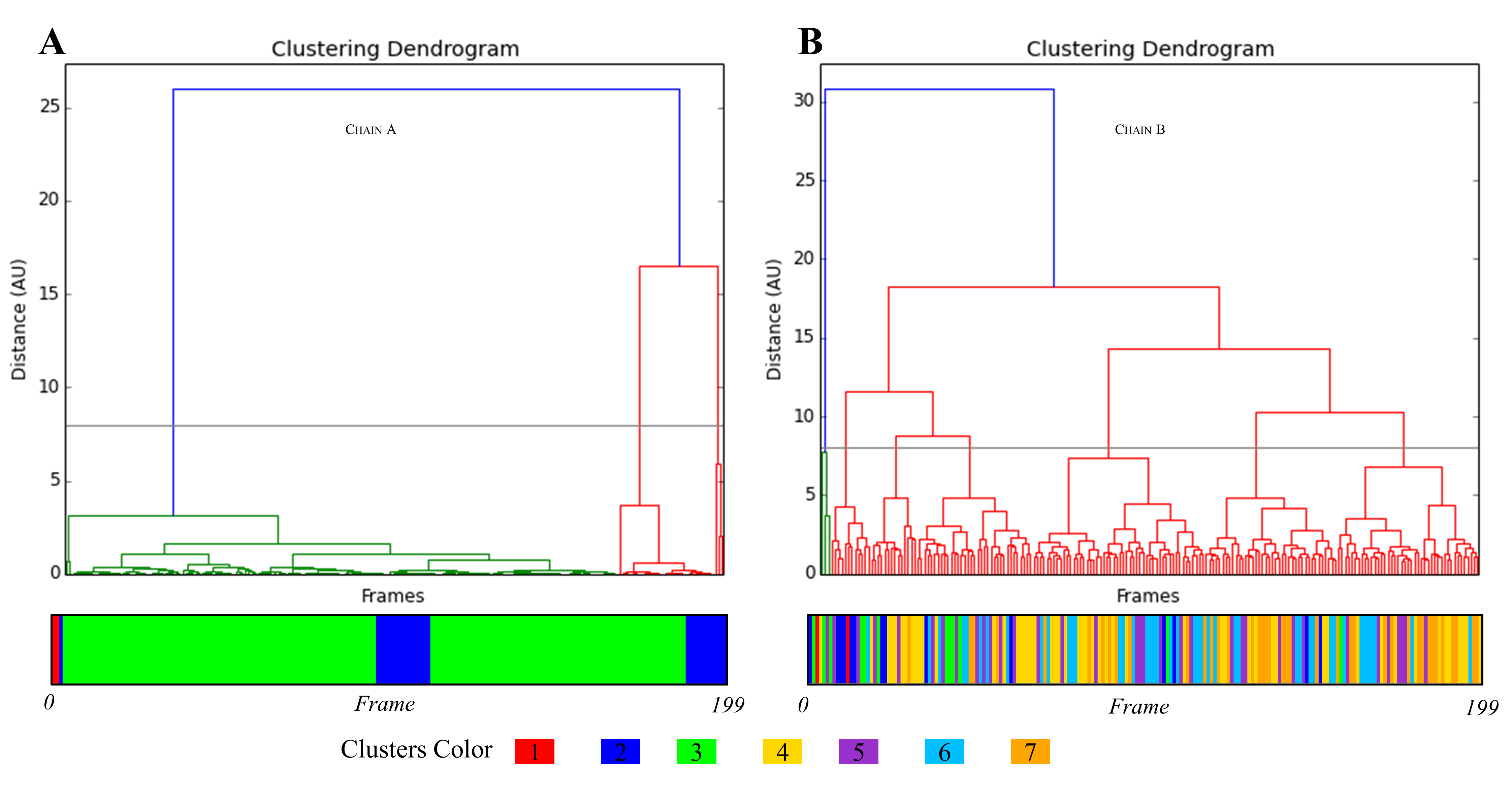

Supplement: S1 Fig — Clustering of N-terminal arm simulated annealing endpoints (A) for chain A and (B) for chain B. Top, clustering dendograms. The cutoff used to generate clusters from hierarchical classification is represented by a gray bar (8 for both chains). Bottom, each successive frame is assigned its cluster color. Graphics were generated with the TrajectoryClustering program (available at https://github.com/tubiana/TrajectoryClustering). (TIF) [file pone.0182056.s001.tif]

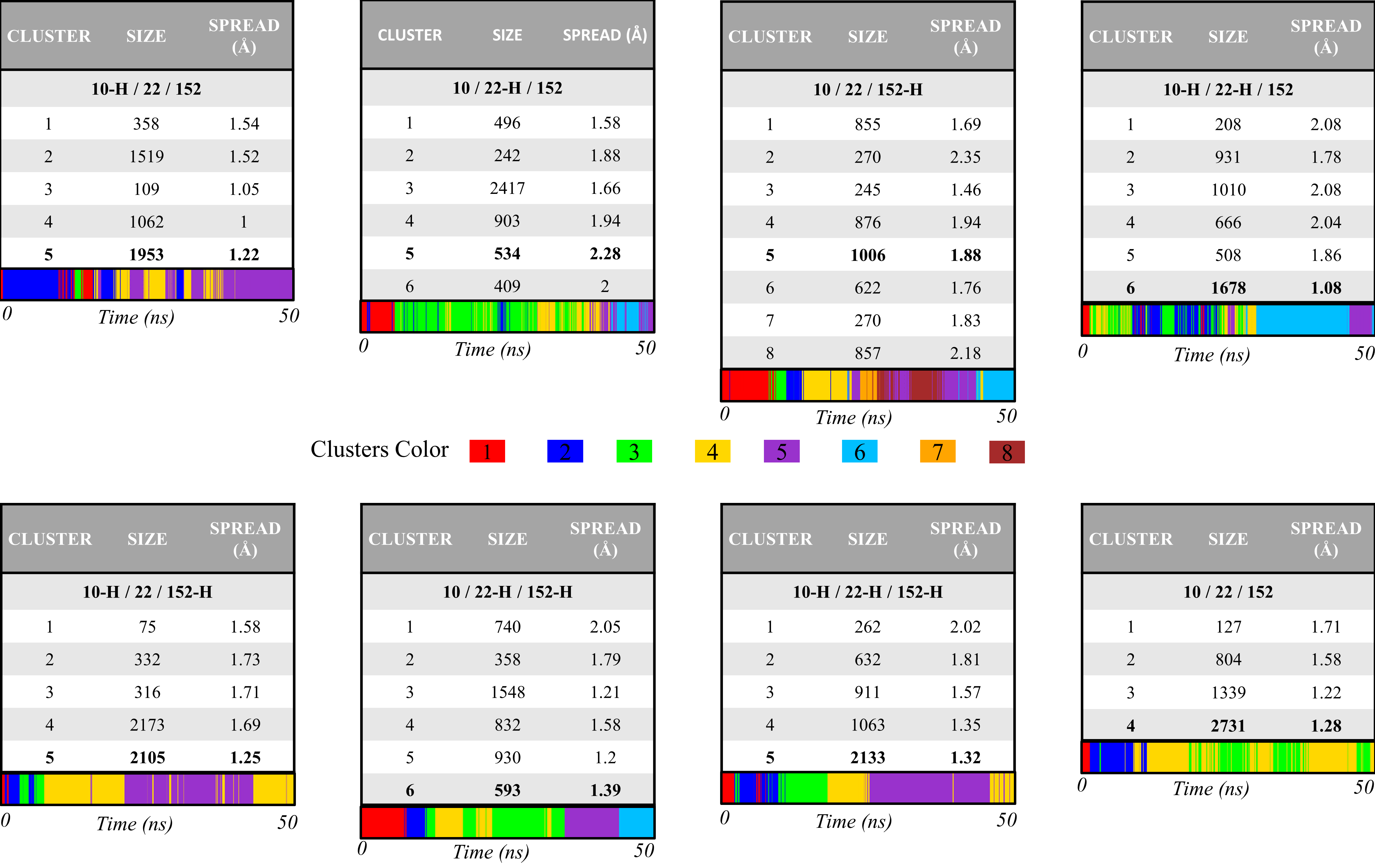

Supplement: S2 Fig — For every cluster, the size (number of frames in the cluster) and the spread (average rmsd between all cluster’s frames) are given. The number of the cluster depends on its order of appearance in the trajectory. A color code was attributed to each cluster. (TIF) [file pone.0182056.s002.tif]

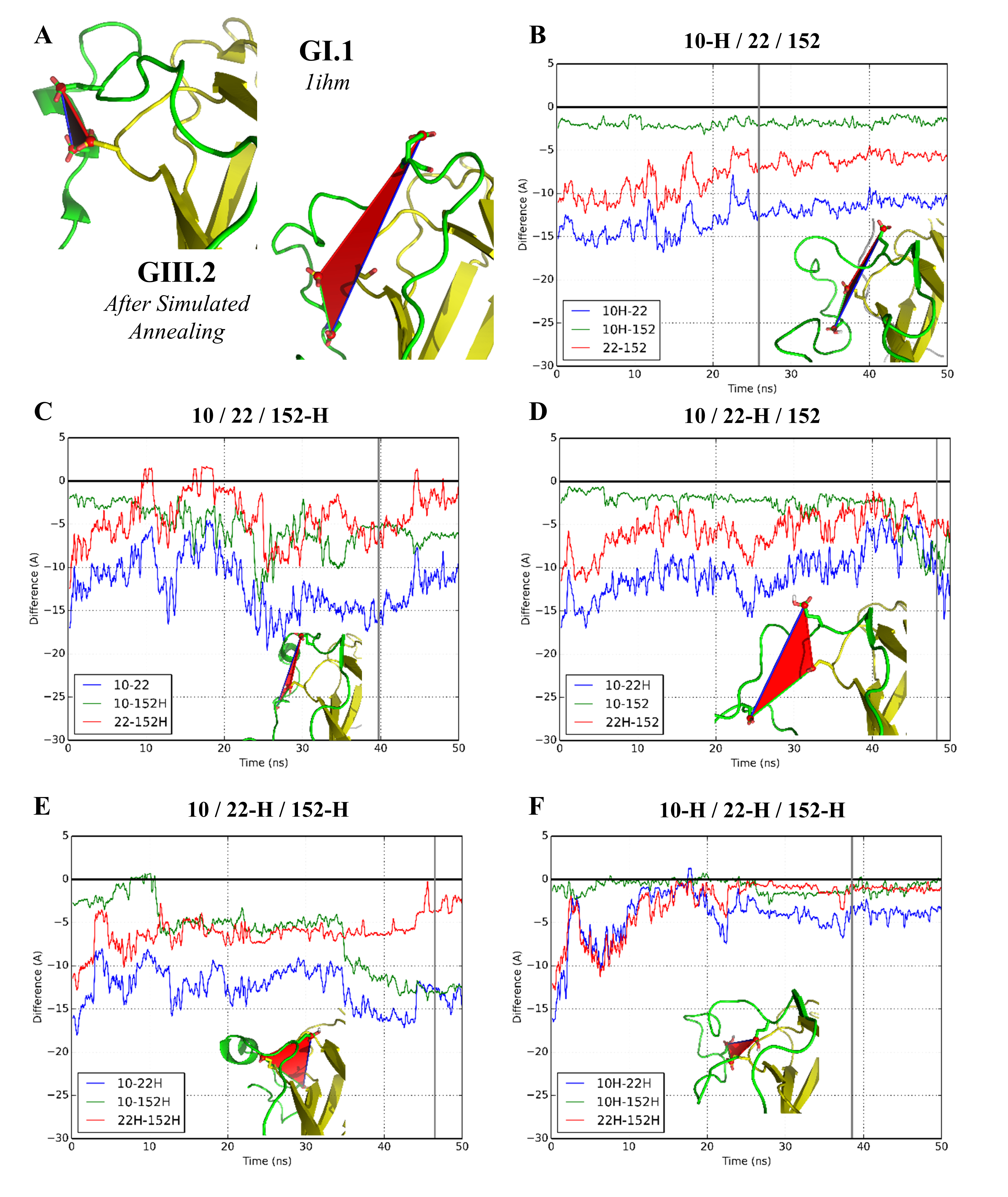

Supplement: S3 Fig — As for Fig 3, (A-F) Evolution of the difference distances between D10, E22 and E152. A value of 0 corresponds to restoration of the initial distance in the GI.1 structure while a negative value indicates a larger distance (see text for details). The illustrations represent the geometry of a representative frame according to the clusterisation of each trajectory (S2 Fig). The gray vertical line indicates the frame from which the picture originates. (TIF) [file pone.0182056.s003.tif]

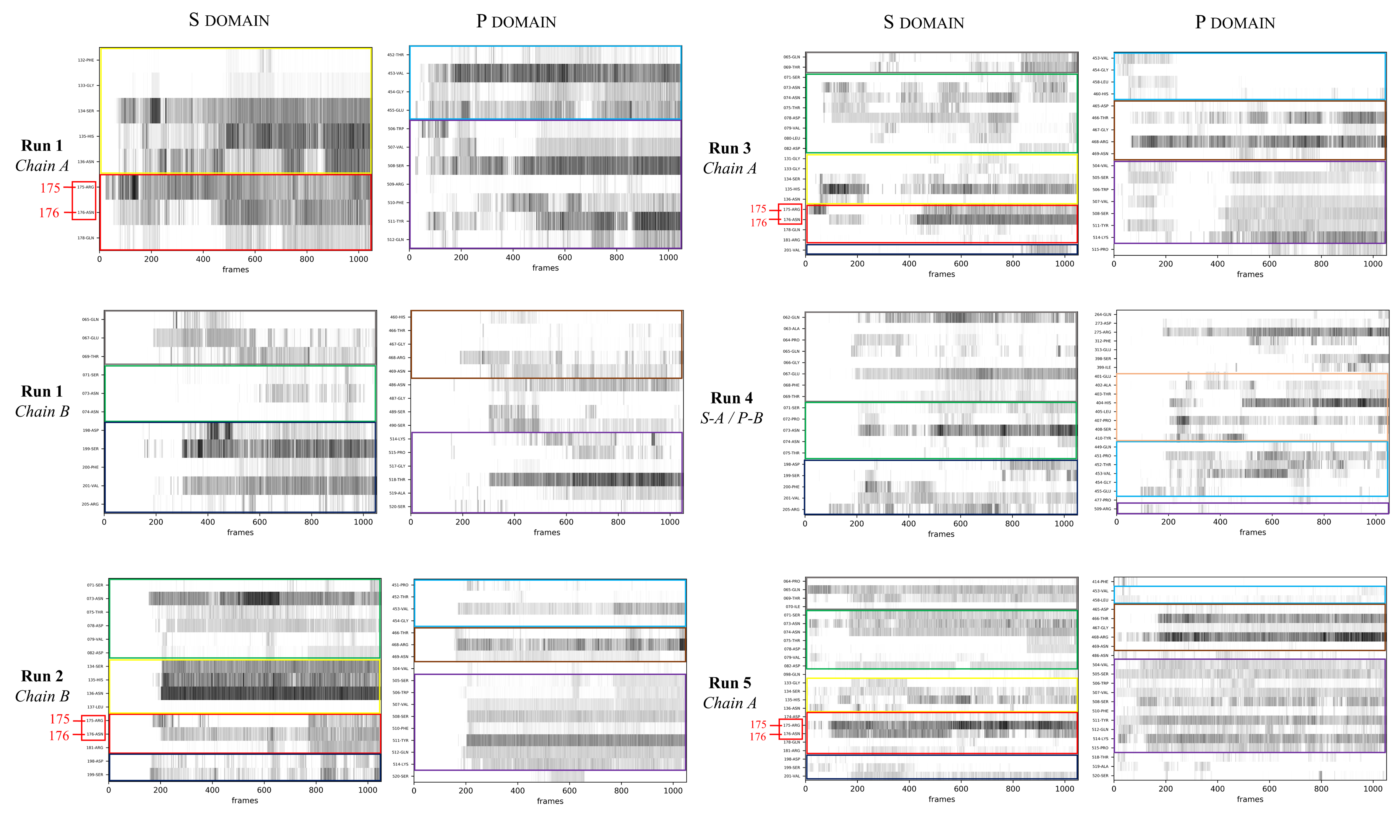

Supplement: S4 Fig — Residues inside a rectangle with the same colour belong to a single segment as defined in Fig 6. Interactions were sampled every 100 ps with VMD [31] and graphics made with matplotlib [32]. (TIF) [file pone.0182056.s004.tif]

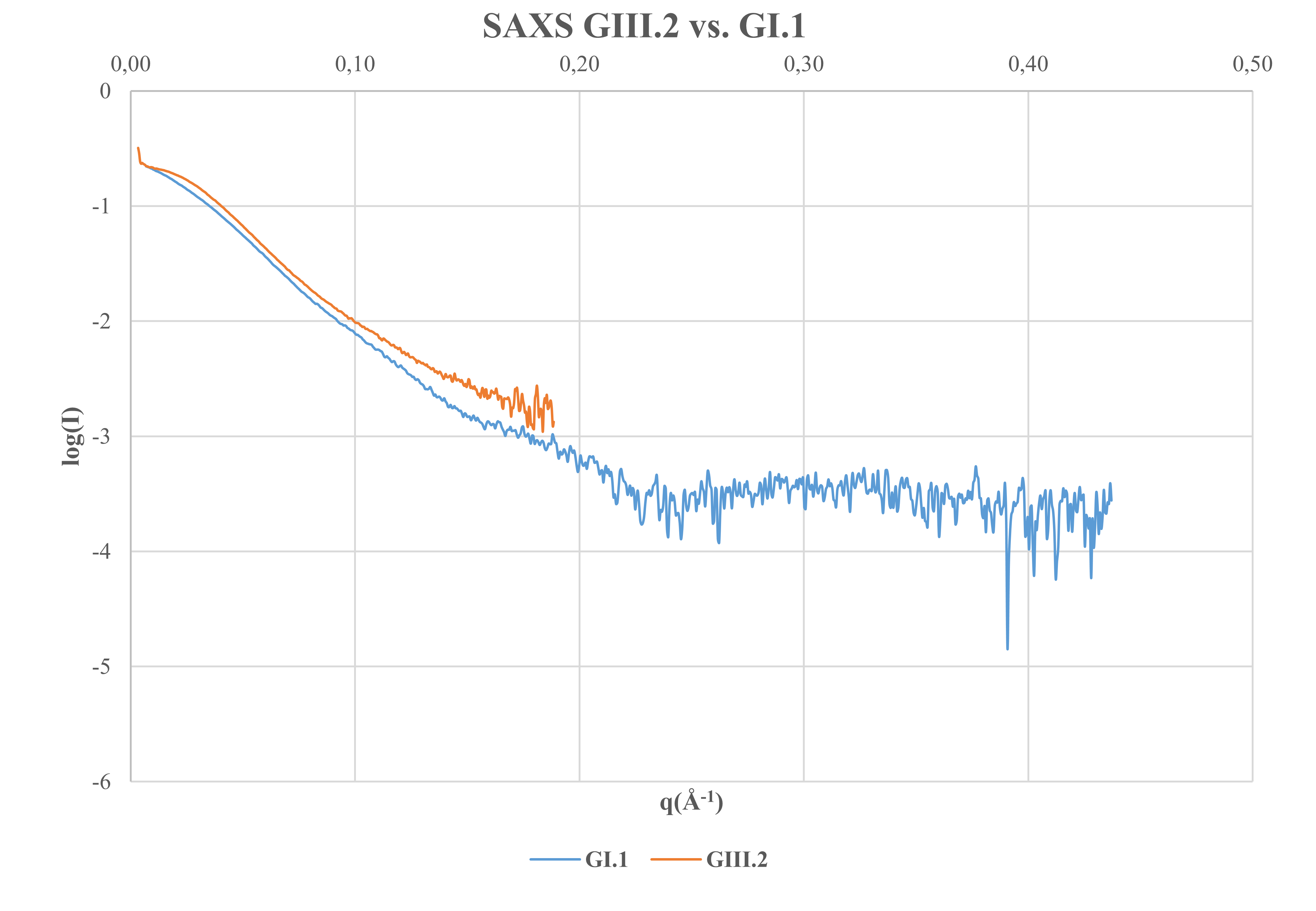

Supplement: S5 Fig — (TIF) [file pone.0182056.s005.tif]

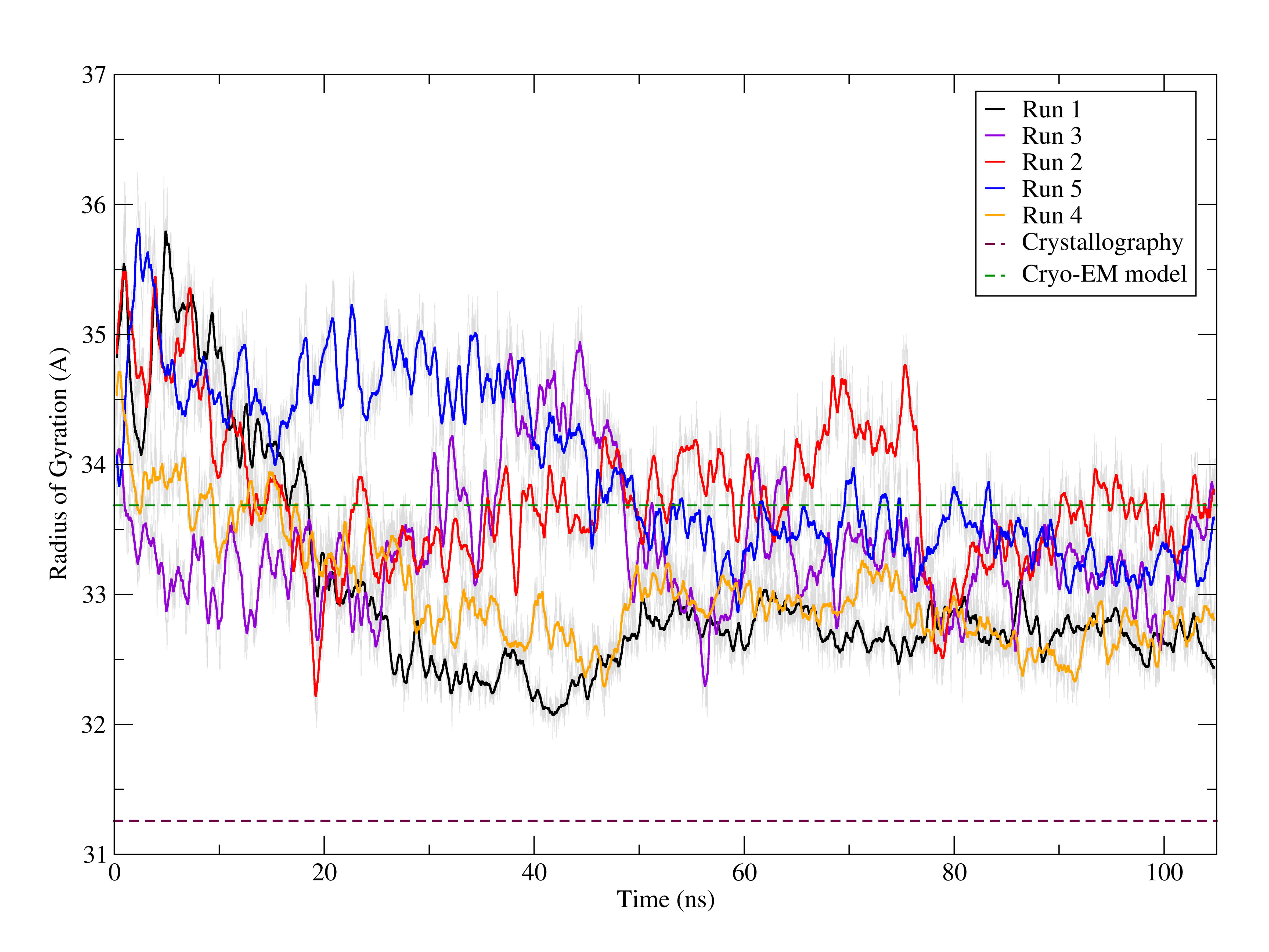

Supplement: S6 Fig — Values for the crystallographic model and our cryo-EM derived model (without termini) are indicated. (TIF) [file pone.0182056.s006.tif]
